# Mechanism of antimicrobial activity of honeybee (*Apis mellifera*) venom on Gram-negative bacteria: *Escherichia coli* and *Pseudomonas* spp.

**DOI:** 10.1186/s13568-021-01214-8

**Published:** 2021-04-09

**Authors:** Izlem Haktanir, Maria Masoura, Fani Th Mantzouridou, Konstantinos Gkatzionis

**Affiliations:** 1 School of Chemical Engineering, University of Birmingham, Edgbaston, Birmingham, B15 2TT UK; 2 Laboratory of Food Chemistry and Technology, Department of Chemistry, Aristotle University of Thessaloniki, 541 24 Thessaloniki, Greece; 3 Department of Food Science and Nutrition, School of the Environment, University of the Aegean, Metropolite Ioakeim 2, 81400 Myrina, Lemnos, GR Greece

**Keywords:** Apitoxin, Antimicrobial mechanism, Metabolic reduction, Membrane integrity, Cell morphology

## Abstract

Honeybee venom (Apitoxin, BV), a secretion substance expelled from the venom gland of bees, has being reported as antimicrobial against various bacterial species; however, the mechanism of action remains uncharacterized. In this study, the antibacterial activity of BV was investigated on hygiene indicator *Escherichia coli* and the environmental pathogen and spoilage bacterial species, *Pseudomonas putida* and *Pseudomonas fluorescens*. An array of methods was combined to elucidate the mode of action of BV. Viability by culture on media was combined with assessing cell injury with flow cytometry analysis. ATP depletion was monitored as an indicator to metabolic activity of cells, by varying BV concentration (75, 225and 500 µg/mL), temperature (25 $^\circ \complement$ and 37 $^\circ \complement$), and time of exposure (0 to 24 h). Venom presented moderate inhibitory effect on *E. coli* by viability assay, caused high membrane permeability and significant ATP loss where the effect was increased by increased concentration. The viability of *P. putida* was reduced to a greater extent than other tested bacteria at comparable venom concentrations and was dictated by exposure time. On the contrary, *P. fluorescens* appeared less affected by venom based on viability; however, flow cytometry and ATP analysis highlighted concentration- and time-dependent effect of venom. According to Transmission Electron Microscopy results, the deformation of the cell wall was evident for all species. This implies a common mechanism of action of the BV which is as follows: the cell wall destruction, change of membrane permeability, leakage of cell contents, inactivation of metabolic activity and finally cell death.

**Supplementary Information:**

The online version contains supplementary material available at 10.1186/s13568-021-01214-8.

## Key points

- Application of BV antimicrobial activity on food spoilage bacterial species was observed.
- Effect of exposure time and BV concentration were driven by species.
- Bacterial cell wall and plasma membrane are putative targets of the BV.

## Introduction

Honeybee venom (BV, Apitoxin) is secreted from venom gland of worker honeybees and it is one of the products of apiculture among others such as honey, propolis, bee wax (Bogdanov 2017; Massaro et al. 2015). BV is a complex substance containing water (88%) and a mixture of peptides, enzymes, amino acids and other components (Additional file 1: Table S1). BV is known to have been used in medicine in the treatment of various diseases, since the time of ancient civilisations (Ali 2012). Currently, BV immunotherapy products attained approval for marketing in many countries such as Bulgaria (Melivenon), Germany (Forapin), Slovakia (Virapin), Canada (Venex), New Zealand (Nectar Balm) (Kokot et al. 2011; Li et al. 2013). Likewise, there is ongoing research on medical applications of BV for asthma, arthritis, Parkinson's disease, Alzheimer's disease (Ali 2012; Socarras et al. 2017; Fratini et al. 2017) and treatment of human cancer cells (Hu et al. 2006; Ip et al. 2012; Jo et al. 2012; Jang et al. 2003; Liu et al. 2013). Despite concerns related to allergenicity and biogenic amine content (Additional file 1: Table S1), there are commercially available products for antiwrinkle facial treatment formulated with BV (e.g., Apiven (France), Manuka Doctor (New Zealand), Rodial (UK)). Although BV biological activity has attracted interest in medical and cosmetic applications, use in food is considerably less than other bee-products such as honey, bee pollen and propolis and was limited to use as a nutrient ingredient, for example in honey. Concerning previous studies, BV presents the potential to act as a natural antimicrobial in food applications.

One of the well evidenced properties of BV and its main components is its antimicrobial activity against bacteria, fungi (Perumal Samy et al. 2007; Al-Ani et al. 2015; Memariani and Memariani 2020), parasites (Adade et al. 2013), and viruses (Uddin et al. 2016). The reported antimicrobial activities of venom and its main components (i.e., melittin and Phospholipase A_2_ (PLA_2_)) against bacterial strains were comprehensively reviewed as part of this study and are listed in Additional file 1: Table S2. Studies have demonstrated the antimicrobial activity of BV against both Gram-positive and Gram-negative species. The Minimum Inhibitory Concentration (MIC) for Gram positive strains ranges from 200 µg/mL to 8 µg/mL for the most sensitive species *Bacillus subtilis* (Al-Ani 2015; Zolfagharian et al. 2016). On the other hand, Gram negative bacterial species appear more resistant to BV (MIC 60 to > 500 µg/mL) (Al-Ani et al. 2015). Leandro et al. (2015) compared BV antimicrobial activity to melittin and PLA_2_ against oral pathogens *Streptococcus salivarius, S. sobrinus, S. mutans, S. mitis, S. sanguinis, Lactobacillus casei*, and *Enterococcus faecalis* by the concentration up to 400 µg/mL: the activity of melittin presented twice the activity of BV against tested bacteria (4 to 40 µg/mL) while PLA_2_ was effective against only *L. casei* at > 400 µg/mL. No synergistic activity of PLA_2_ and melittin was observed. Similarly, antimicrobial activity of melittin was found against Streptococcal and Staphylococcal strains including methicillin-resistant *S. aureus* (MRSA) strains, while PLA_2_ did not exhibit any effect or synergetic activity on the cell viability (Choi et al. 2015). Recently, the synergetic activity of melittin and low power ultrasonication has been proposed as more inhibitory against *Listeria monocytogenes* compared to that for each antimicrobial agent separately (Wu and Narsimhan 2017). To the best of our knowledge, from the mechanistic point of view, PLA_2_ hydrolyses phospholipids at low rate for prolonged periods, so indirectly disrupts the cell membrane of bacteria (Banks and Shipolini 1986). In addition, melittin, the major compound of BV, is known for being responsible for most of the antimicrobial, anti-allergic, anti-inflammatory, and anti-cancer effects of BV (Hu et al. 2006; Dong et al. 2015; Woods et al. 2017; Lee et al. 2019) because of Antimicrobial peptides (AMPs) properties (Adade et al. 2013). As described in previous studies, melittin increases cell permeability and integrates into phospholipid bilayers in low concentrations, and forms pores in the cell membrane in high concentrations which causes the release of Ca^2+^ ions or breaks phospholipid groups (Fennell et al. 1968; Shipolini 1984; Adade et al. 2013; Wu et al. 2016; Socarras et al. 2017). However, the outer membrane of Gram-negative bacteria obstructs penetration of melittin into the cytoplasmic membrane (Shai 2002; Al-Ani et al. 2015).

Although, the composition and effectiveness of BV against several bacteria are well reported, the investigation of the associated mechanism of action is limited to the role of melittin. In this study, different methods were combined to elucidate the antimicrobial activity and mode of action of BV against the Gram-negative *Escherichia coli* and for the first time *Pseudomonas putida* and *Pseudomonas fluorescens*. The effect of BV was investigated by culture on media and was correlated with cell membrane damage by assessing cell injury with flow cytometry (FC) analysis. ATP depletion was monitored as an indicator to metabolic activity of cells and changes on the cell membrane were further analysed by transmission electron microscopy (TEM). Activity of BV on bacterial species was tested on stationary phase at different temperature (25 °C and 37 °C) and time of exposure (0 to 24 h).

## Materials and methods

### Materials and samples

Two batches of commercial freeze-dried *Apis mellifera* BV samples obtained by electrostimulation were used in this study, namely “BV-1” (Henan-Senyuan Biological Technology Co Ltd, China) and “BV-2” (Citeq biologics, Netherlands). Melittin ($\ge 85$% purity) was purchased from Sigma-Aldrich (UK). Nutrient agar (Oxoid Ltd., CM003), Nutrient broth (Oxoid Ltd., CM0001) and Phosphate-buffered saline (PBS) were supplied by Fisher Scientific (United Kingdom). Culture medium Luria–Bertani (LB) broth (Miller, L3152) and two stains, bis-(1,3-dibutylbarbituric acid) trimethine oxonol (DiBAC_4_(3)) and propidium iodide (PI), were purchased from Sigma-Aldrich (UK). HPLC grade water and acetonitrile (ACN) were from Chem-Lab (Belgium). Trifluoroacetic acid (TFA) was from Acros organics (Belgium). All other common reagents were of the appropriate purity from various suppliers.

### Microbial cultures

Three Gram-negative bacterial strains *E. coli* K-12, MG1655 (ATCC 47,076), *P. putida* (ATCC 700,008), and *P. fluorescens* (NCIMB 9046) were maintained on nutrient agar petri dishes at 4 °C. Cultures were grown at 37 °C for *E. coli* in LB broth and *P. putida* in Nutrient broth*,* and at 25 °C for *P. fluorescens* in nutrient broth for 24 h shaking at 150 rpm. Cell cultivation yielded mid-stationary phase population of *E. coli, P. putida* and *P. fluorescens* with a concentration of approximately 10^8^ CFU (Colony Forming Units)/mL. After centrifugation ($\text{11 200 x g}$, 10 min), cells were washed in Phosphate-buffered solution (PBS) twice and re-suspended in 1 mL of PBS before use in antimicrobial assays.

### Viability analysis by culture

One milligram of each of the BV samples was used to prepare working solutions of 150, 450, and 1000 µg/mL in deionized water. For each strain, 100µL aliquots of cell suspension was mixed with 100µL of 150, 450 and 1000 µg/mL of BV working solutions or deionized water (control) in 96-well plates and incubated for 24 h at 25 °C and 37 °C shaking at 150 rpm. Bacterial viability was assessed at different time points of incubation (0, 4 and 24 h). Each sample was serially diluted in PBS buffer and plated on nutrient agar plates using the Miles and Misra technique (Miles et al. 1938). Each dilution was plated on nutrient agar and incubated at 37 °C for *E. coli* and *P. putida*, and at 25 °C for *P. fluorescens* for 24 h. Following, the viable bacterial counts (CFU/mL) were determined.

### Assessment of cell membrane integrity by FC analysis

Treated bacterial cultures were stained by adding 4 µl/mL of PI and DiBAC_4_(3) and incubated in the dark for 5 min. Stained cultures were analysed using an Attune Nxt, Acoustic Focusing Cytometer (Thermo Fisher Scientific, Singapore). Cells were excited with a blue laser at 488 nm, and the emitted fluorescence was detected through a 400 nm band-pass filter for both dyes. The trigger was set for the green fluorescence (550 nm) channel and data acquired on dot plot of forward-scatter versus side scatter. Volumetric counting had an experimentally determined quantification limit of 10,000 events. All samples were performed in triplicate and the data was analysed using the Invitrogen Attune Nxt Software (Version 2.7).

### Monitoring of cell metabolic activity by ATP analysis

Based on the results of viability and FC, the applied concentration of 75 and 500 µg/mL BV at 0 and 24 h were considered for testing metabolic activity. BacTiter-Glo™ Microbial Cell Viability Assay (Promega, USA) and a CLARIOstar Luminometer (BMG Labtech, Germany) were used for the quantitation of the ATP present in bacterial cell culture. The changes in metabolic activity of treated cells were assessed based on the reduction of relative light unit (RLU) in relation to control cells. The BacTiter-Glo™ Microbial Cell Viability Assay was prepared according to manufacturer guidelines. A 100µL aliquot from each treated-cell culture was mixed with an equal volume of BacTiter-Glo™ reagent in triplicate and incubated for 5 min at 150 rpm shaking. After incubation, the luminescence of samples was immediately measured with a Luminometer and analysed using MARS data analysis software.

### TEM analysis of microbial cells treated with BV.

The changes of bacterial cell structure after BV treatment were observed with a JEOL 1400 transmission electron microscope with Morada Soft Imaging system. For each strain, cell suspension was prepared (Sect. 2.2), mixed with 1000 µg/mL of BV solutions or deionized water (control) in 1 mL microcentrifuge tube (1:1) and incubated for 24 h at 25 °C, shaking at 150 rpm. Following, bacterial cells were centrifuged at 1372×*g* for 10 min. The supernatant was discarded, and the pellet was washed twice by re-suspension in PBS followed by centrifugation. The cells were then fixed by suspending the pellet in 2.5% glutaraldehyde (in 0.1 M phosphate buffer, pH 7.4) and stored at 4 °C for 1 h. After primary fixation, the samples were washed with PBS. Cells were post-fixed with 1% osmium tetroxide for 1 h and washed briefly with distilled water. The post-fixed specimens were dehydrated in a graded ethanol series (twice in 50, 70, 90, 100%, 100% dried Alcohol for 15 min each). The specimens were further treated with propylene oxide twice each for 15 min as a transitional fluid and then embedded in resin. The polymerisation of the resin to form specimen blocks was accomplished in an oven at 60 °C for 16 h. Ultrathin sections were cut with a diamond knife using an ultramicrotome and then mounted on bare copper grids. They were stained with 2% uranyl acetate and lead citrate, followed by examination with the electron microscope.

### RP-HPLC analysis of melittin

BV-1 and BV-2 dry samples were suitably diluted in HPLC-grade water. The resulting aqueous solutions (150 μg/mL) were filtered through a 0.45 μm PTFE filter (Waters, Milford, MA) before RP-HPLC analysis conducted as described by Rybak-Chmieleska and Szczesna (2004). The HPLC system was equipped with a LC-20AD pump (Shimadzu, Kyoto, Japan) and a SPD-10AV UV–VIS detector (Shimadzu). Separation was achieved on a chromatographic column C18 (L x I.D., 250 mm × 4.6 mm, 5 μm particle size) (BioBasic, Thermo Scientific, UK). The elution system was consisted of 0.1% TFA in water (Solvent A) and 0.1% TFA in the solution of ACN: water (80:20) (Solvent B). The linear gradient elution for solvent B was 5%—80% (40 min). The flow rate was 1 ml/min (25 °C) and the injection volume 20µL. Peak identification was based on standard available, relative retention time and literature. Quantification of melittin (μg/mL) was performed using external calibration curve (220 nm) and calculated by linear regression analysis.

### Statistical analysis

All measurements and treatments were performed in triplicate (N = 3). Statistical comparisons of the mean values carried out by one-way ANOVA, followed by Student’s t-test using the SPSS 20.0 software (SPSS Inc., Chicago, IL). Results were considered statistically significant at *p* < 0.05.

## Results

### Effect of BV on viability of the bacteria

**Fig. 1:**
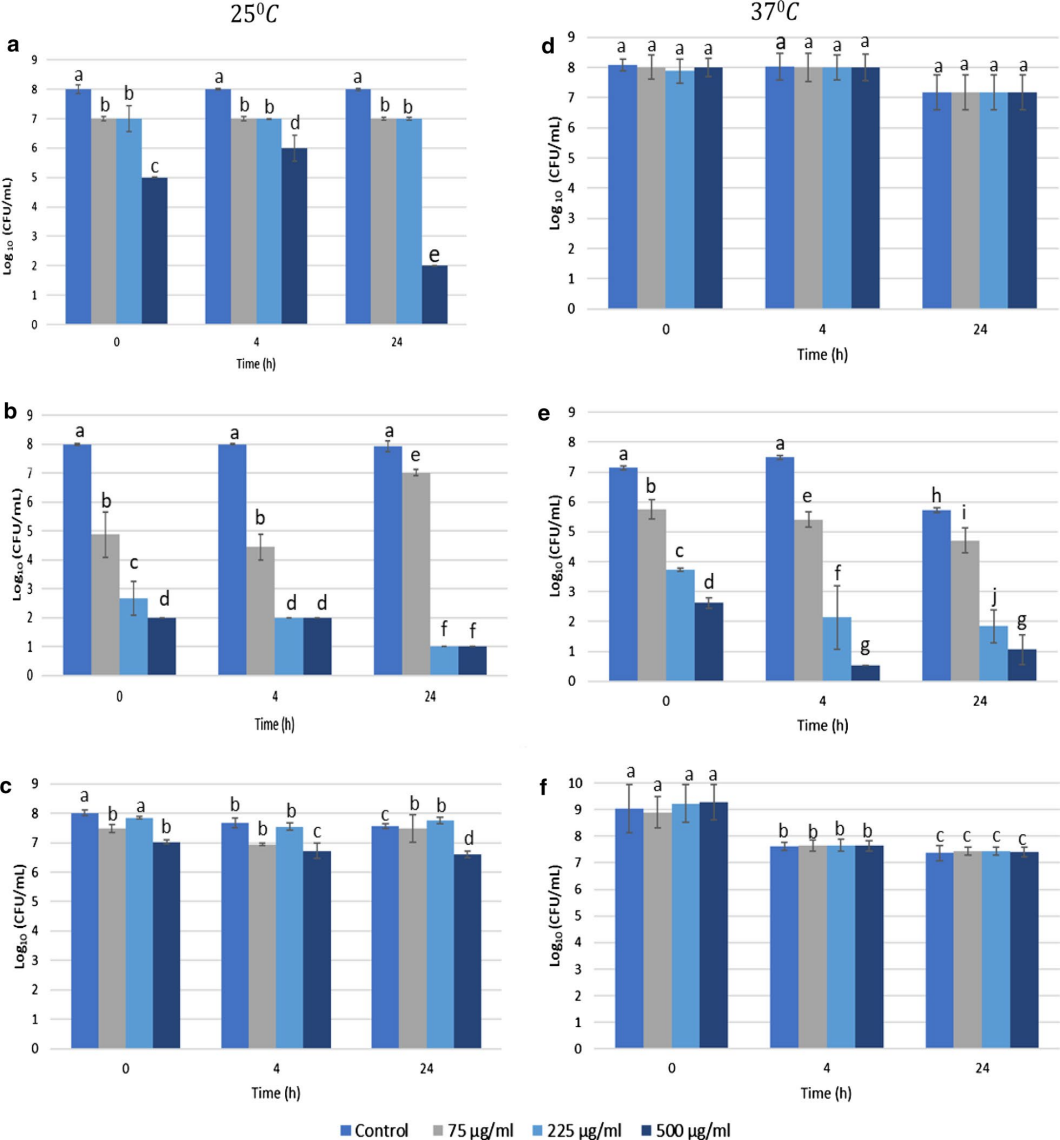
Viability (CFU/mL) of **a**, **d** *E. coli* MG1655, **b**, **e** *P. putida* ATCC 700,008 and **c**, **f** *P. fluorescens* NCIMB 9046 incubated with BV-1 for 0, 4 and 24 h at 25 °C (Left) and 37 °C (Right). Error bars represent the standard deviation (sd) of the mean value (N = 3)

**Fig. 2:**
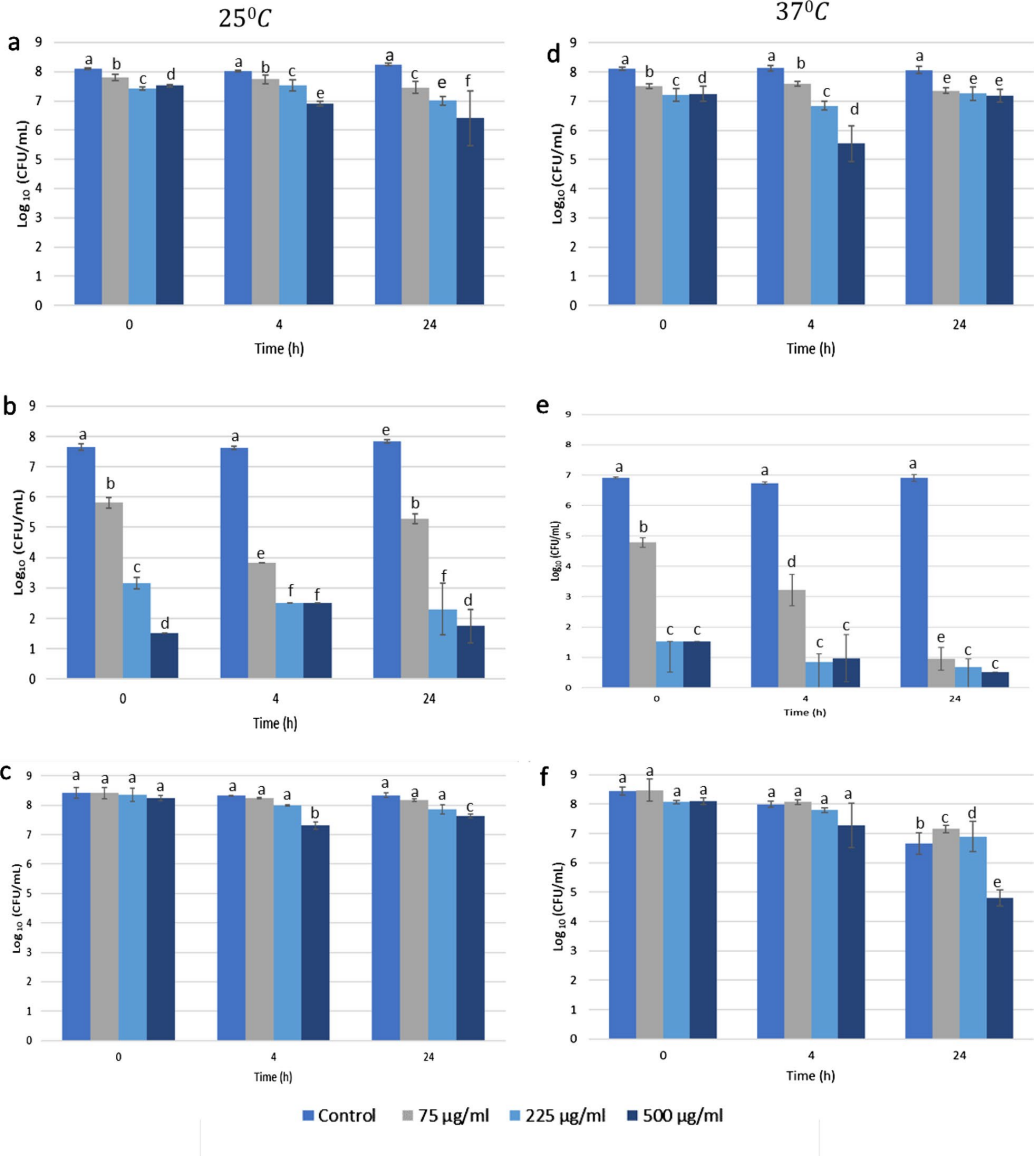
Viability (CFU/mL) of **a**, **d** *E. coli*, MG1655, **b**, **e** *P. putida*, ATCC 700,008 and **c**, **f** *P. fluorescens*, NCIMB 9046 in CFU/mL incubated with BV-2 for 0, 4 and 24 h at 25 °C (Left) and 37 °C (Right). Error bars represent the standard deviation (sd) of the mean value (N = 3)

The effects of samples BV-1 and BV-2 on cells were comparable (Figs. 1, 2). The effect of BV on *E. coli* cells varied based on the conditions of treatment. *E. coli* treated with BV-1 at 25 °C presented a decrease in viability. This was less affected by increase in BV concentration for BV-2. Variation between BV samples can be explained by qualitative and quantitative differences in composition recorded by HPLC profiles of aqueous solutions of BV-1 and BV-2 (150 μg/mL) at 220 nm (Additional file 1: Figure S1), For example, the 1.3-fold higher concentration of melittin in solution of BV-2 compared with that in solution of BV-1 (62 vs 47.5 $\mathrm{\mu g}/\mathrm{mL}$) could greatly affect their bactericidal activity.

Significant inhibition was observed when treating the cells with high concentration of BV (500 µg/mL) and for extended time (24 h) (Figs. 1, 2). *P. putida* was significantly affected by exposure time to BV regardless of temperature. The viability decreased proportionally to the increase of BV concentration (*p* < 0.05). However, 225 and 500 µg/mL of BV did not differ significantly in effect after 4 h of exposure for both samples (Figs. 1, 2), suggesting adaptation of treated *P. putida* cells. In contrast, *P. fluorescens* appeared to be unaffected by BV regardless of concentration and exposure time or temperature.

### Effect of BV on bacterial membrane integrity

**Fig. 3:**
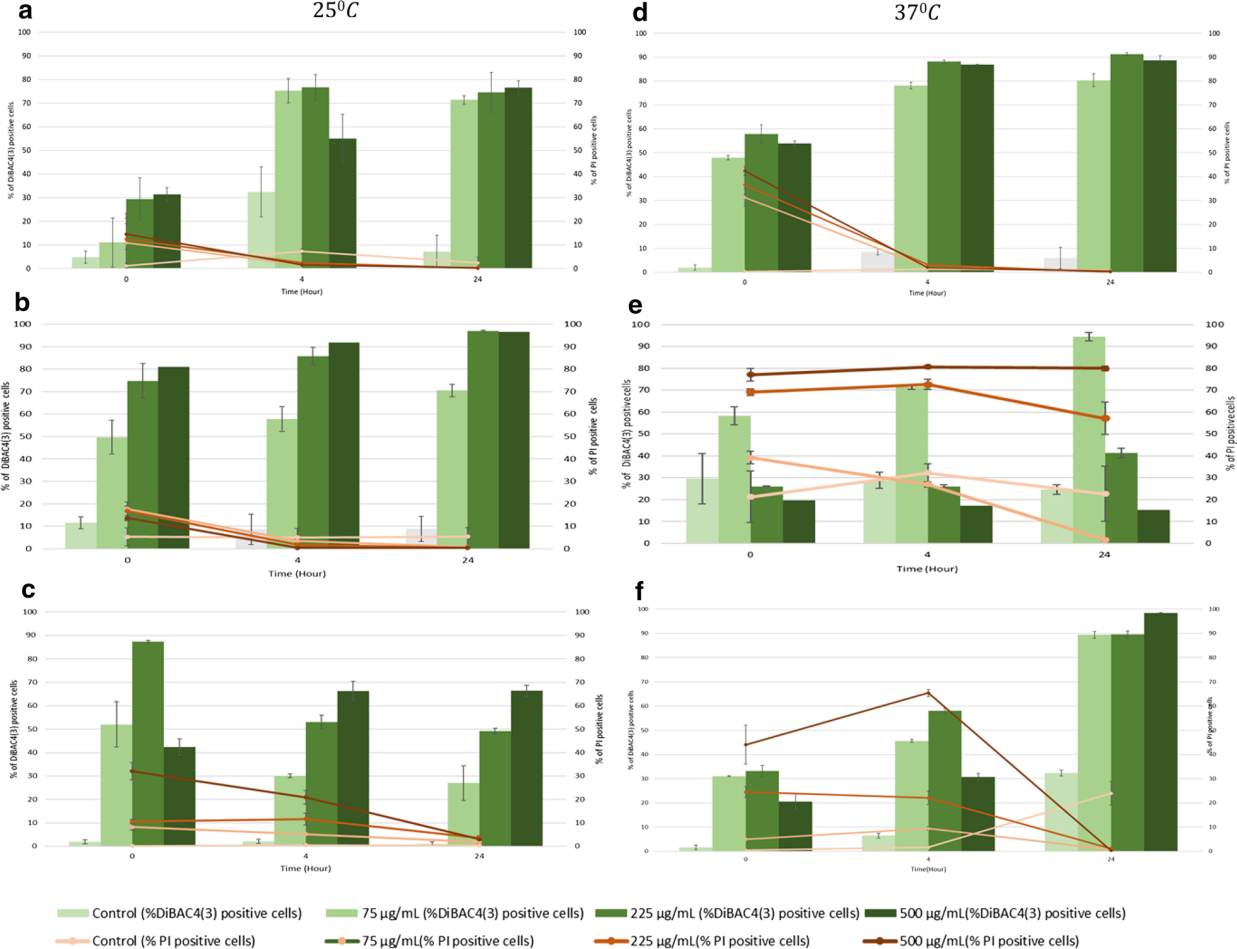
Percentage of PI positive and DiBAC_4_(3) positive bacterial cells measured by flow cytometry after BV-1 treatment at 0, 4 and 24-h incubation at 25 °C (Left) and 37 °C (Right). **a**, **d** *E. coli*, MG1655, **b**, **e** *P. putida*, ATCC 700,008 and **c**, **f** *P. fluorescens*, NCIMB 9046. Error bars represent the standard deviation (sd) of the mean value (N = 3)

**Fig. 4:**
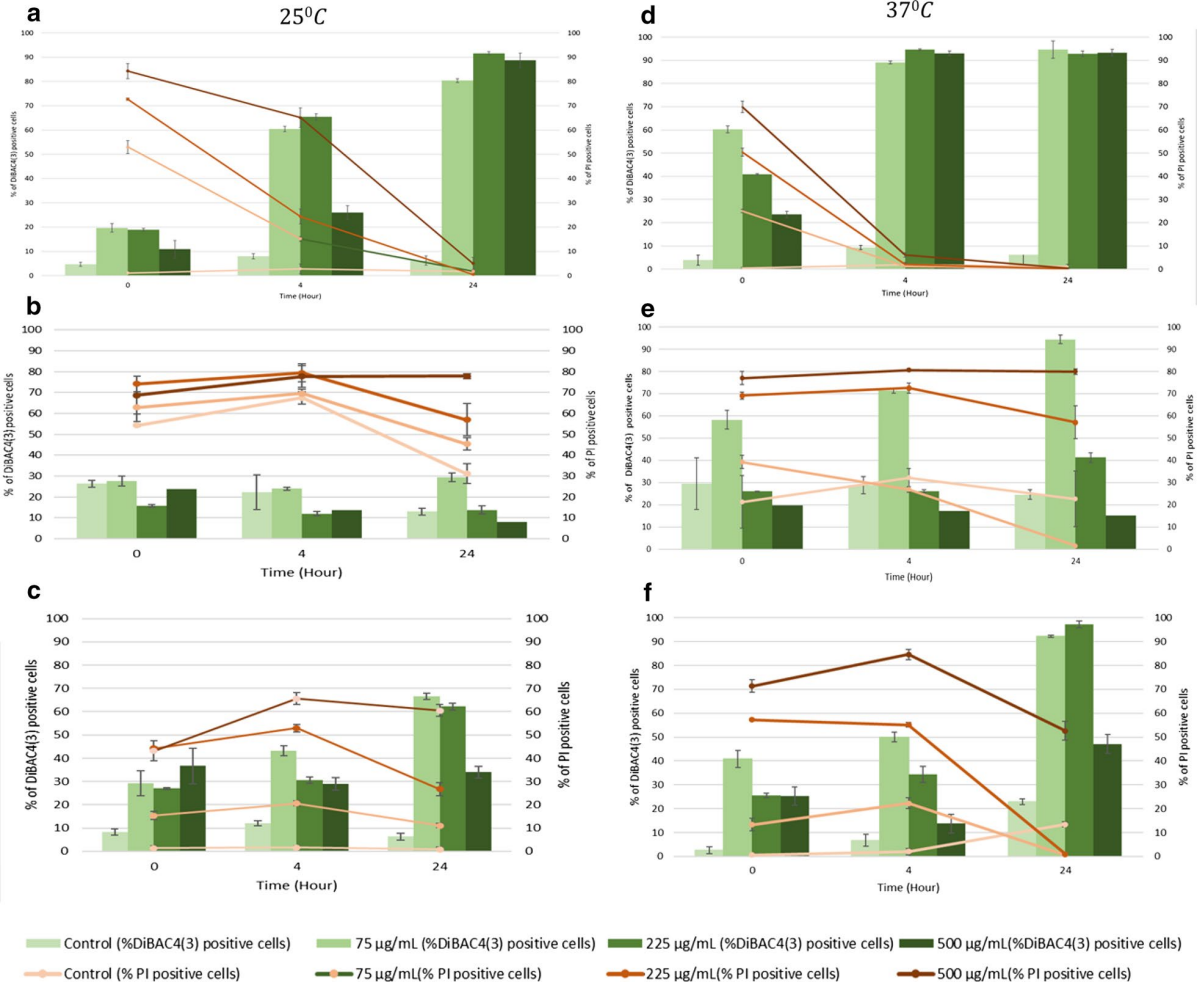
Percentage of PI positive and DiBAC_4_(3) positive bacterial cells measured by flow cytometry after BV-2 treatment at 0, 4 and 24-h incubation at 25 °C (Left) and 37 °C (Right). **a**, **d** *E. coli*, MG1655, **b**, **e** *P. putida*, ATCC 700,008 and **c**, **f** *P. fluorescens*, NCIMB 9046. Error bars represent the standard deviation (sd) of the mean value (N = 3)

FC analysis was employed to study bacterial injury in response to BV treatment. For treated bacteria, the percentage of PI-positive cells was significantly greater at all time points (0, 4 and 24 h) than the untreated cells at 25 °C and 37 °C (*p* < 0.05) (Figs. 3, 4). Despite no evidence of detrimental decrease in cell viability in analysis by culture, for same conditions of treatment, *E. coli* presented significant increase of PI-positive cells percentage, especially for the case of BV-2 (Fig. 4), suggesting bactericidal effect at time zero. Following 4 h of BV treatment at 75 and 225 µg/mL, DiBAC_4_(3)-positive cells significantly increased by 70%, representing suspended injury of *E. coli* treated cells; however, increasing BV concentration to 1000 µg/mL did not increase further the number of DiBAC_4_(3)-positive cells (Figs. 3, 4).

Aligned with the responses observed in viability tests, *P. putida* cell membrane was significantly damaged by exposure time. BV-1 presented a significant increase in percentage of PI-positive cells compared to untreated at 37 °C, whereas the number of DiBAC_4_(3)-positive cells were over 50% at 25 °C at time zero. However, DiBAC_4_(3)-positive cells significantly increased over 24 h regardless of temperature (Fig. 3). The PI-positive cells increased proportionally to the increase in BV-2 concentration at time zero, whereas DiBAC_4_(3)-positive cells increased over 24-h, except for treated cells at 500 µg/mL (Fig. 4).

*P. fluorescens* viability by culture seemed to be unaffected by BV regardless of concentration, exposure time or temperature; however, the DiBAC_4_(3) positive cells (Fig. 3) and PI-positive cells (Fig. 4) were initially observed for 500 µg/mL. Following 24 h of BV treatment, injury of cells and damage of membrane were increased proportionally to the increase in BV concentration.

### Effect of BV treatment on metabolic activity

ATP-depletion in treated cells showed a strong effect of BV on metabolic activity. The ATP level of *E. coli* was significantly reduced (33%) when treated with 500 µg/mL BV and around 30% at 24-h (Table 1). Similarly, treated cells of *P. putida* presented significant ATP reduction during incubation. The percentage of metabolically active cells was less than 10% following 24-h BV treatment. In the case of *P. fluorescens,* ATP in treated cells presented a reduction by 20% with 500 µg/mL.

**Table 1:** Percentage of metabolically active cells after bee venom treatment at 0 and 24-h at 25 °C

Time (hour)	BV-1				BV-2			
	0 h		24 h		0 h		24 h	
Bacterial species	75 µg/mL	500 µg/mL	75 µg/mL	500 µg/mL	75 µg/mL	500 µg/mL	75 µg/mL	500 µg/mL
*E. coli*	100	33	29	20	85	36	28	28
*P. putida*	100	100	78	43	100	100	12	2
*P. fluorescens*	115	128	99	27	138	127	85	12

### Analysis of cell morphological changes

**Fig. 5:**
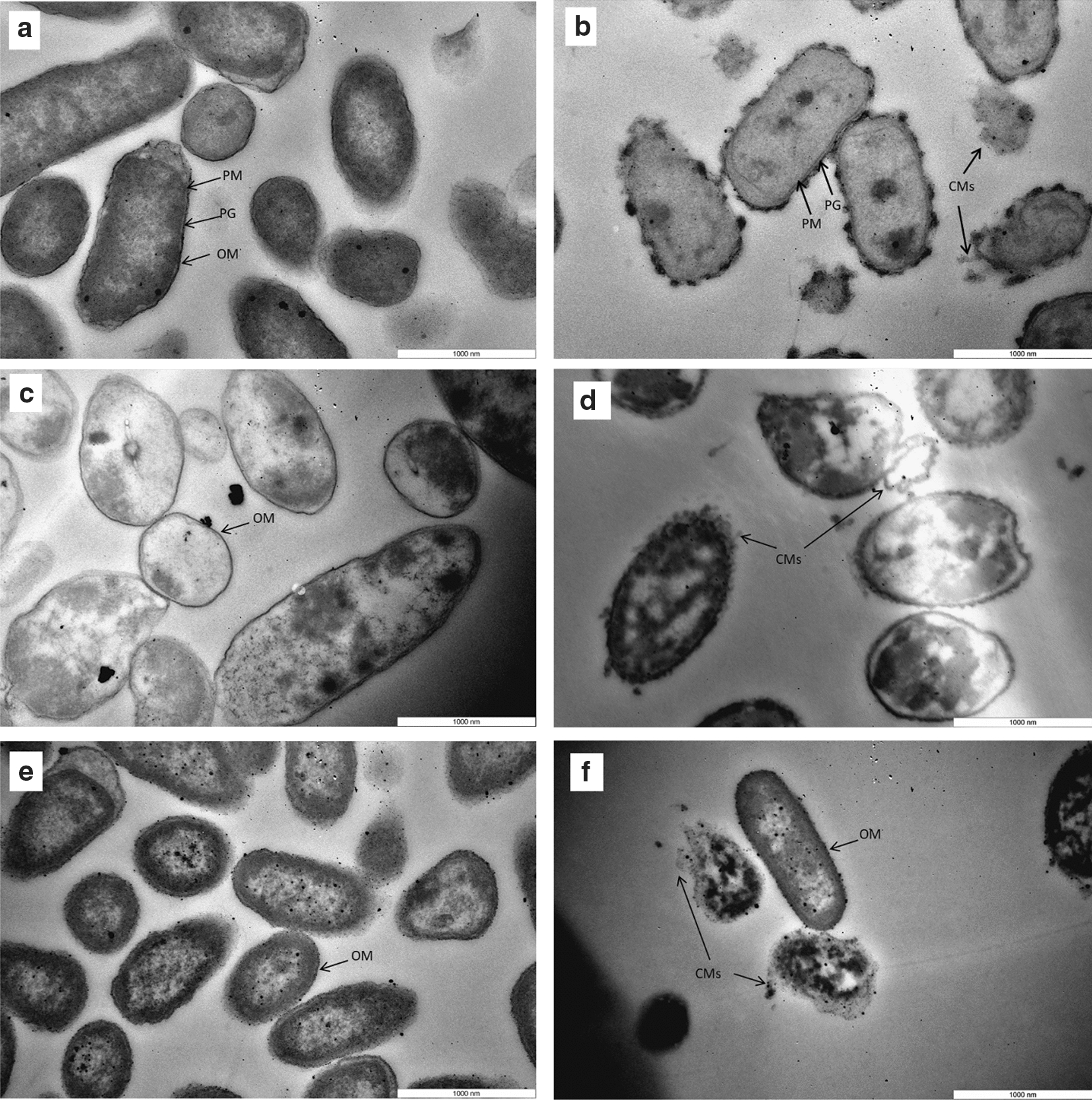
Morphological changes of *E. coli strain, MG1655,* control (**a**) and treated (**b**), *P. putida strai*n, ATCC 700,008 control (**c**) and treated (**d**), *P. fluorescens strain,* NCIMB 9046 control (**e**) and treated (**f**) after 24-h BV-2 treatment (500 $\mu g/mL$ at 25 °C observed by TEM (magnification 50 K). Control cells were prepared incubated with de-ionised water. *OM* outer membrane, *PG* peptidoglycan layer, *PM* plasma membrane, *CMs* cytoplasmic materials

TEM was employed in order to visualise possible morphological changes in the wall and internal structure of bacterial cells. In the absence of BV, the bacterial cell membrane appeared intact with high-density cytoplasm for all species (Fig. 5). Upon exposure of *E. coli* cells to BV for 24 h, membrane disruption was observed, and the leaked cytoplasmic material was found to be formed around the membrane. *P. putida* cell wall and the cytoplasmic membrane showed uneven envelope, lysis of membrane integrity and leakage of intracellular contents, resulting in cytoplasmic vacuolation. The phospholipid bilayer of *P. fluorescens* cells was seriously deformed and the cell membrane was heavily damaged resulting to cytoplasmic leakage. Unlike other species, there were cells displaying intact structures and high-density of cytoplasm.

## Discussion

BV has been shown to exert potent activity in microorganism against tested Gram-negative bacteria. Moreover, it was demonstrated that BV will be more effective if it is delivered in a manner that ensures optimum conditions of time and concentration. In this study, the variation in the number of viable cells treated with BV was found to be primarily driven by bacterial species. *E. coli, P. fluorescens* and *P. putida* presented different patterns in reduction of viability, for the same concentrations of BV. Therefore, these findings are consistent with previous reports, the activity of BV against *E. coli* between 100 µg/mL and 500 µg/mL (Al-Ani et al. 2015) while 1800 µg/mL of BV was found the minimum concentration for inhibition (Hegazi et al. 2017). The effect of BV on *P. putida* and *P. fluorescens* have been studied for the first time in this study, hence, comparison of results is not available. Surendra et al. (2011) has previously reported the antimicrobial activity of BV against *P. aeruginosa* to be concentration dependent, and the MIC was found 2400 µg/mL by Hegazi et al. (2017). Similarly, the bacteriostatic activity of BV against *P. fluorescens* and *P. aeruginosa* (Al-Ani et al. 2015) was found 500 µg/mL. Moreover, the viability of *P. putida* was concluded in this study as most sensitive bacteria against BV at tested concentrations, followed by *E. coli* and *P. fluorescens,* suggesting, regardless of genera, species dependent BV activity which was also concluded in Choi et al. (2015).

In many cases of antibacterial agents, the target was the cell membrane, which is crucial for maintaining growth/survival by isolating the intracellular material and energy balance. Hence, the effectiveness of a preservative is related to the damage to the cell membrane structure and disturbance of the function of enzyme system for the growth inhibition of bacteria (Yao 2012). It seems that BV affects membrane integrity and the plasma membrane potential of *E. coli* cells in association to significant loss of viability. In addition, the adaptation of treated *P. putida* cells was observed at 75 µg/mL BV over 24 h. Therefore, the lethal effect of BV appeared to depend on exposure time above 75 µg/mL. *P. fluorescens* distinctly presented sublethal stress behaviour, resulting injury and less metabolic activity at 24 h.

The formation of pores and their size is acknowledged as crucial for the bacterial recovery death. Previous studies on Gram positive cells suggested that the effect of BV on cell membrane permeability is associated to melittin by forming of pores on the cell wall, and a property of AMPs (Wu & Narsimhan 2017). In a study conducted by Wu et al. (2016), the effect of melittin was observed by TEM comprised damage and pore formation in the cell membrane of Gram-positive *S. aureus* followed by increased cell permeabilization through the cytoplasmic membrane. However, the outer membrane of Gram-negative bacteria, which contains lipopolysaccharides (LPS), obstructs penetration of melittin into the cytoplasmic membrane (Shai 2002; Al-Ani et al. 2015). To the best of our knowledge, the second main compound, PLA_2_, enzymatically hydrolyses phospholipids at low rate for prolonged periods which indirectly disrupts the cell membrane of Gram-negative bacteria (Banks and Shipolini 1986). Therefore, the antimicrobial mechanisms of action of melittin could not associated as the mechanism of BV on Gram negative bacterial cells.

The present study confirmed that cell wall and membrane disruptions increase membrane permeability. Following 24 h BV treatment, the leaked cytoplasmic materials were found to be formed around all tested cells. The phospholipid bilayer of bacteria was deformed the cell membrane was heavily damaged and the shape of some cells became irregular. Cytoplasm was not evenly distributed, resulting in cytoplasmic vacuolation. Hence, the microbial cell growth was inhibited by BV. However, the observation of intact structure *P. fluorescens* cells also suggested the resistance against BV which is consistent with the results obtained from culture analysis, FC and ATP analysis. Although the complete mechanism of action of BV against bacteria has not been fully elucidated yet, together, the data of the present study demonstrated for the first time, to the best of our knowledge, BV may be used as a promising natural antimicrobial agent on Gram-negative species from pharmaceutical to food applications.

BV consists of a large number of peptides. Therefore, activity similar to AMPs could be expected in food matrices and processes, in terms of water solubility, thermostability, tolerance to high or low ionic strength and pH values. In this study, BV antimicrobial activity was investigated under the optimal temperatures (25 °C and 37 °C) for the growth of the target microorganisms common in food applications under a range of low concentrations (75, 225 and 500 µg/mL). The results indicated that the activity of BV was evident in most tested conditions. Yet, BV has not been exploited as a natural food preservative at industrial scale, and research is rather limited. The challenge would be to ensure BV stability and activity in the presence of intrinsic food properties and environmental conditions that are known to affect bacterial growth and food safety. Studies in solid model systems have shown that food structure protects immobilized or surface attached cells due to the development of acid tolerance and susceptibility to inhibitory agents such as lactic acid or antimicrobial agents (Boons et al. 2013; Lobete et al. 2015; Noriega et al. 2010). Also, BV is considered a rich source of biogenic amines such as histamine (Barkiene et al. 2020; Official Journal of the European Union 2005), a fact that could limit its threshold in food applications.

## Supplementary Information

**Additional file 1: Table S1.** Composition of dry honeybee venom, (Shipolini 1984; Pucca et al. 2019). **Table S2.** Summary of studies on antimicrobial activity of honeybee venom against bacteria. **Figure S1.** HPLC chromatograms of melittin standard aqueous solution (50 μg/mL) (a) BV-1 (b) and 45 BV-2 (c) aqueous solutions (150 μg/mL) at 220 nm. Peak (*): melittin. Detection was at 220 nm 46 (Chromatographic conditions as in Materials and Methods section).

## Data Availability

The data that support the findings of this study are available from the corresponding author upon reasonable request.
