# Supplementary material for: Mechanism of antimicrobial activity of honeybee (Apis mellifera) venom on Gram-negative bacteria: Escherichia coli and Pseudomonas spp
Source: AMB Express. 2021 Apr 9;11:54. doi: 10.1186/s13568-021-01214-8 (PMC8035396; doi:10.1186/s13568-021-01214-8)

Mechanism of antimicrobial activity of honeybee (*Apis mellifera*) venom on Gram-negative bacteria: *Escherichia coli* and *Pseudomonas* spp.

Izlem HAKTANIR[a,*], Maria MASOURA[a], Fani Th MANTZOURIDOU[b], Konstantinos GKATZIONIS[a,c,*]

a School of Chemical Engineering, University of Birmingham, Edgbaston, Birmingham B15 2TT, United Kingdom

b Laboratory of Food Chemistry and Technology, Department of Chemistry, Aristotle University of Thessaloniki, 541 24 Thessaloniki, Greece

c Department of Food Science and Nutrition, School of the Environment, University of the Aegean, Metropolite Ioakeim 2, GR 81400 Myrina, Lemnos, Greece

*Corresponding author:

Konstantinos Gkatzionis, email: kgkatzionis@aegean.gr; telephone: +306972567325

Izlem Haktanir, email: izlemhaktanir@gmail.com ; telephone:+4407460805150

Table S1. Composition of dry honeybee venom, (Shipolini, 1984; Pucca et al., 2019)

Class of molecules	Compound	Amount (% of venom, dry weight)
**Enzymes**	Phospholipase A_2[b]	10-12
	Hyaluronidase[a]	1,5-2
**Peptides**	Melittin[a]	40-50
	Apamin	3
	Mast Cell Degranulating Peptide (MCDP)	2
**Amines**	Histamine[c]	0.6-1.6
	Dopamine	0.13-1
**Carbohydrates**	Glucose	0.7
	Fructose	0.9
**Amino acids**	γ-aminobutyric acid and β-aminoisobutyric acid	1
**Lipids**		5
**Minerals**	P, Ca, Mg	3-4
a: minor allergen / potent allergen, b: major allergen, c: biogenic amine		

Table S2: Summary of studies on antimicrobial activity of honeybee venom against bacteria.

	**Bacteria**						
**Component**	**Gram positive**	**Gram negative**	**Strains**	**Assay**	**MIC (µg/mL)**	**MBC (µg/mL)**	**References**
Bee venom		*Ac. baumannii BAA*	*ATCC 747*	MM	30	60	Al-Ani et al., 2015
	B. subtilis		*ATCC 6051*	MM	8	10	
		Bur. mallei	*RTCC 2375*	ADM			Zolfagharian et al., 2016
		Bur. pseudomallei	*RTCC 2375*	ADM			
		E. coli	*ATCC 35218*	BDM	ND		Hegazi et al., 2017
			ATCC 25922	MM	60	60	Al-Ani et al., 2015
			SR 200756	MM	100	200	
			KL 206272	MM	200	200	
				ADM	NA	NA	Perumal Samy et al., 2007
		E. coli O157:H7	*ATCC 35150*	MM	100	200	Al-Ani et al., 2015
			ATCC 25923				Zolfagharian et al., 2016
		En. aerogenes	-	ADM	NA	NA	Perumal Samy et al., 2007
	Ent. casseliflavus		*ATCC 700327*	MM	10	30	Al-Ani et al., 2015
	Ent. feacalis			BDM	ND		Hegazi et al., 2017
			ATCC 29212	MM	100	200	Al-Ani et al., 2015
			ATCC 4082	MM	20	-	Leandro et al., 2015
	Ent. feacalis VanB		*ATCC 51299*	MM	200	500	Al-Ani et al., 2015
	Ent. VRE		*ATCC 902291*	MM	200	500	
			ATCC 102105	MM	200	500	

		ATCC 902316	MM	200	500	
	K. pneumoniae	*ATCC 27736*	BDM	ND		Hegazi et al., 2017
		ATCC 700603	MM	30	60	Al-Ani et al., 2015
		ATCC 800877	MM	500	500	
		KL 206436	MM	500	500	
	K. oxytoca	*ATCC 700324*	MM	500	500	
Lact. caseai		*EMCC 1093 T*	BDM	ND		Hegazi et al., 2017
		ATCC 11578	MM	20	-	Leandro et al., 2015
Lis. monocytogene ne			BDM	ND	-	Hegazi et al., 2017
Myc. smegmatis		*ATCC 19420*	MM	200	200	Al-Ani et al., 2015
Myc. fortuitum		*ATCC 6841*	MM	100	100	
Myc. phlei		*ATCC 11758*	MM	800	800	
	Pr. mirabilis	-	ADM	-	-	Perumal Samy et al.,2007
	Pr. vulgaris	-	ADM	-	-	
	P. aeruginosa	*ATCC 27853*	BDM	ND	-	Hegazi et al., 2017
			MM	500	>500	Al-Ani et al., 2015
						Zolfagharian et al., 2016
		KL 206466	MM	500	>500	Al-Ani et al., 2015
		KL 206444	MM	>500	>500	
		-	ADM	-	-	Perumal Samy et al., 2007
	Sal. choleraesuis	*ATCC 554*	MM	500	>500	Al-Ani et al., 2015
	Sal. typhimurium	*ATCC 25923*				Zolfagharian et al., 2016
	Sh. flexneri	*ATCC 29903*	MM	60	100	Al-Ani et al., 2015
Staph. aureus		*ATCC 25923*	BDM	ND	-	Hegazi et al., 2017

						Zolfagharian et al.,2016
			MM	10	30	Al-Ani et al., 2015
		ATCC 29213	MM	60	200	
		USA300 (LAC)	MM	0.78	ND	Choi et al., 2015
		Newman	MM	0.78	ND	
		MW2	MM	1.56	ND	
		MRSA1	MM	3.12	ND	
		MRSA2	MM	1.56	ND	
		ISP4790	MM	6.25	ND	
		MU50	MM	6.25	ND	
		ATCC 6538P		17	ND	Kokot et al., 2009
		-	ADM	23.2±1.09	-	Perumal Samy et al., 2007
Staph. aureus BAA		*ATCC 977*	MM	30	60	Al-Ani et al., 2015
Staph. aureus MRSA		*ATCC 1042*	MM	60	100	
		ATCC 106804	MM	60	200	
		ATCC 106188	MM	60	100	
		ATCC 106091	MM	60	200	
Staph. epidermidis		*ATCC 14990*	MM	60	100	
Staph. saprophyticus		*ATCC 15305*	MM	10	10	
Strep. agalactiae		*ATCC 27956*	MM	40	40	
		CNCTC 10/84	MM	6.25	ND	Choi et al., 2015
Strep. bovis		*NEM 760*	MM	1.56	ND	
Strep. epidermidis		*RP62a*	MM	0.78	ND	
Strep. gordonii		*M99*	MM	6.25	ND	
Strep. mitis		*ATCC 49452*	MM	40	-	Leandro et al., 2015
Strep. mutans		*EMCC 1815T*	BDM	ND	-	Hegazi et al., 2017
		ATCC 25175	MM	20	-	Leandro et al., 2015

	Strep. oralis		*ATCC 35037*	MM	100	100	Al-Ani et al., 2015
	Strep. pneumonia		*TIGR4*	MM	3.12	-	Choi et al., 2015
	Strep. pyogenes			BDM	ND	-	Hegazi et al., 2017
			ATCC 12344	MM	100	200	Al-Ani et al., 2015
	Strep. salivarius		*ATCC 25975*	MM	20	-	Leandro et al., 2015
	Strep. sanguinis		*ATCC 10556*	MM	30	-	
	Strep. sobrinus		*ATCC 33478*	MM	40	-	
	Strep. thermophilus		*ATCC 19258*	MM	30	60	Al-Ani et al., 2015
Melittin		*Ac. baumannii BAA*	*ATCC 747*	MM	30	30	Al-Ani et al., 2015
	B. subtilis		*ATCC 6051*	MM	6	30	
		E. coli	*ATCC 25922*	MM	30	60	
			SR 200756	MM	30	60	
			KL 206272	MM	30	60	
		E. coli O157:H7	*ATCC 35150*	MM	30	60	
	Ent. casseliflavus		*ATCC 700327*	MM	8	20	
	Ent. feacalis		*ATCC 29212*	MM	30	100	
			ATCC 4082	MM	6	-	Leandro et al., 2015
	Ent. feacalis VanB		*ATCC 51299*	MM	50	100	Al-Ani et al., 2015
	Ent. VRE		*ATCC 902291*	MM	50	100	
	Lact. casei		*ATCC 11578*	MM	4	-	Leandro et al., 2015
		K. pneumoniae	*ATCC 700603*	MM	100	300	Al-Ani et al., 2015
			ATCC 800877	MM	100	300	
		K. oxytoca	*ATCC 700324*	MM	60	500	

		P. aeruginosa	*ATCC 27853*	MM	100	100	
			KL 206466	MM	100	100	
			KL 206444	MM	100	100	
	Staph. aureus		*ATCC 25923*	MM	10	30	
			ATCC 29213	MM	6	10	
	Staph. aureus BAA		*ATCC 977*	MM	8	8	
	Staph. aureus MRSA		*ATCC 1042*	MM	100	30	
			ATCC 106804	MM	30	50	
			ATCC 106188	MM	10	50	
			ATCC 106091	MM	30	50	
	Staph. epidermidis		*ATCC 14990*	MM	10	30	
	Staph. saprophyticus		*ATCC 15305*	MM	10	30	
	Strep. pyogenes		*ATCC 12344*	MM	10	30	
	Strep. oralis		*ATCC 35037*	MM	200	200	
	Strep. agalactia		*ATCC 27956*	MM	30	30	
	Strep. mitis		*ATCC 49452*	MM	10		Leandro et al., 2015
	Strep. mutans		*ATCC 25175*	MM	40		
PLA$_2$		*E. coli*	-	ADM	NA	NA	Perumal Samy et al., 2007
		En. aerogenes	-	ADM	NA	NA	
		Ent. feacalis	*ATCC 4082*	MM	NA	NA	Leandro et al., 2015
	Lact. casei		*ATCC 11578*	MM	400	-	
		Pr. mirabilis	-	ADM	NA	NA	Perumal Samy et al., 2007
		Pr. vulgaris	-	ADM	NA	NA	
		P. aeruginosa	-	ADM	NA	NA	
	Staph. aureus		-	ADM	13.3±0.83	-	
	Strep. mitis		*ATCC 49452*	MM	NA	NA	Leandro et al., 2015

	Strep. mutans		*ATCC 25175*	MM	NA	NA	

Ac.: *Acinetobacter*	En.: Enterobacter	Lis.: *Listeria*	Sal.: *Salmonella*
B.: *Bacillus*	Ent.: Enterococcus	Myc.: *Mycobacterium*	Sh.: *Shigella*
Bur.: Burkholderia	K.: *Klebsiella*	Pr.: *Proteus*	Staph.: *Staphylococcus*
E.: Escherichia	Lact.: *Lactobacillus*	P.: *Pseudomonas*	Strep.: *Streptococcus*
ADM: Agar Dilution Method	BDM: Broth Dilution Method	MM: Microdilution Method	ND: Not determined

Figure S1. HPLC chromatograms of melittin standard aqueous solution (50μg/mL) (a) BV-1 (b) and BV-2 (c) aqueous solutions (150μg/mL) at 220nm. Peak (*): melittin. Detection was at 220nm (Chromatographic conditions as in Materials and Methods section).

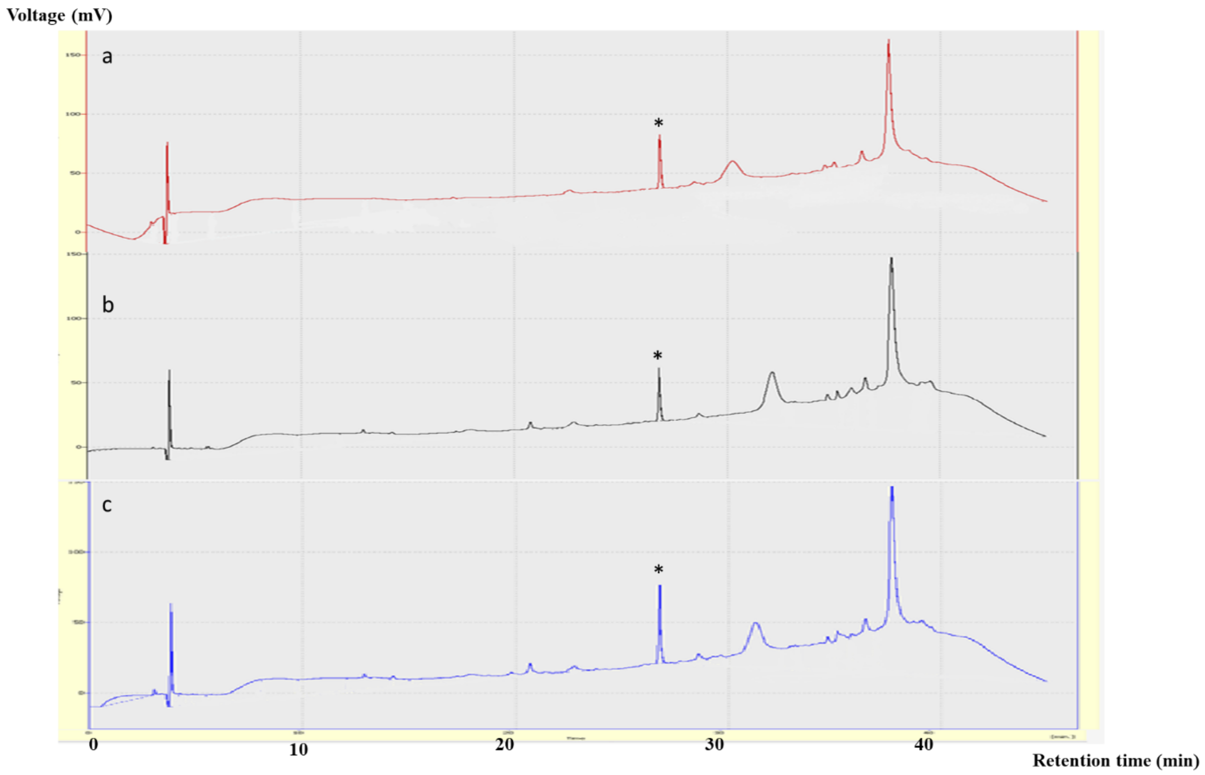

Supplement: Supplementary file 1 — Additional file 1: Table S1. Composition of dry honeybee venom, (Shipolini 1984; Pucca et al. 2019). Table S2. Summary of studies on antimicrobial activity of honeybee venom against bacteria. Figure S1. HPLC chromatograms of melittin standard aqueous solution (50 μg/mL) (a) BV-1 (b) and 45 BV-2 (c) aqueous solutions (150 μg/mL) at 220 nm. Peak (*): melittin. Detection was at 220 nm 46 (Chromatographic conditions as in Materials and Methods section). [file 13568_2021_1214_MOESM1_ESM.pdf]
